# Substantial and sustained reduction in under-5 mortality, diarrhea, and pneumonia in Oshikhandass, Pakistan: evidence from two longitudinal cohort studies 15 years apart

**DOI:** 10.1186/s12889-020-08847-7

**Published:** 2020-05-24

**Authors:** C. L. Hansen, B. J. J. McCormick, S. I. Azam, K. Ahmed, J. M. Baker, E. Hussain, A. Jahan, A. F. Jamison, S. L. Knobler, N. Samji, W. H. Shah, D. J. Spiro, E. D. Thomas, C. Viboud, Z. A. Rasmussen, for the Oshikhandass Diarrhea and Pneumonia Project, Anita Zaidi, Arif Amin Khan, Ahmed Jan, Faheemullah Beg, Saba Wasim, Azad Wali Khan, Israr Ahmed, Musa Rahim, Shamsuddin Mughal, Sher Baz Khan, Shaheen Mughal, Bulbul Jahan, Mumtaz Mughal, Zeenat Keswani, Wilayat Shah, Aftab Mukhi, Farah Hashmani, Faran Sikandar, Sahrish Durrani, Julie Goodwin, Kristen Hulbert, Arif Hussain, Mirza Jibran, Asif Hussain, Meherbano, Mukhi Bano, Nazara Begum, Malika Zadi, Razia Sultana, Zohra Begum, Iqbal Bano, Parveen Bano, Gulshan Jan, Nusrat Jabeen, Dil Roz, Malika Meri, Sajida Parveen, Naseem Bano, Gulab Jan, Zohra Bano, Mobina Bano, Gul Nasreen, Mehtab Bano, Kaniz Fatima, Iqbal Bano, Dil Roz, Nazara, Ghazala, Nasima Begum, Alia Rani, Mehwish Hakeem, Rubina, Sameena, Zevar Jan, Sunaira, Resham Jan

**Affiliations:** 1 Fogarty International Center, National Institutes of Health, Bethesda, MD 20892 USA; 2 Department of Community Health Sciences, Aga Khan University, Stadium Road, Karachi, 74800 Pakistan; 3 Karakoram International University, University Road, Gilgit, Pakistan

**Keywords:** Diarrhea, Pneumonia, Under-5 mortality, Infant mortality, Pakistan, Community-based healthcare

## Abstract

**Background:**

Oshikhandass is a rural village in northern Pakistan where a 1989–1991 verbal autopsy study showed that diarrhea and pneumonia were the top causes of under-5 mortality. Intensive surveillance, active community health education and child health interventions were delivered in 1989–1996; here we assess improvements in under-5 mortality, diarrhea, and pneumonia over this period and 15 years later.

**Methods:**

Two prospective open-cohort studies in Oshikhandass from 1989 to 1996 (Study 1) and 2011–2014 (Study 2) enrolled all children under age 60 months. Study staff trained using WHO guidelines, conducted weekly household surveillance and promoted knowledge on causes and management of diarrhea and pneumonia. Information about household characteristics and socioeconomic status was collected. Hurdle models were constructed to examine putative risk factors for diarrhea and pneumonia.

**Results:**

Against a backdrop of considerable change in the socioeconomic status of the community, under-5 mortality, which declined over the course of Study 1 (from 114.3 to 79.5 deaths/1000 live births (LB) between 1989 and 1996), exceeded Sustainable Development Goal 3 by Study 2 (19.8 deaths/ 1000 LB). Reductions in diarrhea prevalence (20.3 to 2.2 days/ Child Year [CY]), incidence (2.1 to 0.5 episodes/ CY), and number of bloody diarrhea episodes (18.6 to 5.2%) seen during Study 1, were sustained in Study 2. Pneumonia incidence was 0.5 episodes /CY in Study 1 and 0.2/CY in Study 2; only 5% of episodes were categorized as severe or very severe in both studies. While no individual factors predicted a statistically significant difference in diarrhea or pneumonia episodes, the combined effect of water, toilet and housing materials was associated with a significant decrease in diarrhea; higher household income was the most protective factor for pneumonia in Study 1.

**Conclusions:**

We report a 4-fold decrease in overall childhood mortality, and a 2-fold decrease in childhood morbidity from diarrhea and pneumonia in a remote rural village in Pakistan between 1989 and 2014. We conclude that significant, sustainable improvements in child health may be achieved through improved socioeconomic status and promoting interactions between locally engaged health workers and the community, but that continued efforts are needed to improve health worker training, supervision, and the rational use of medications.

**Trial registration:**

Not Applicable.

## Background

Addressing persistent health challenges, such as childhood morbidity and mortality in lower- and middle-income countries, requires sustained intervention, a key component of which is building local engagement and capacity in the community [1, 2]. Despite considerable success in reducing the global burden of childhood pneumonia and diarrhea through, for example, improvements in nutritional status, exclusive breast-feeding, and immunization for pneumonia and the use of oral rehydration and more recently, rotavirus vaccine for diarrhea [3, 4], these two diseases continue to be leading causes of under-5 mortality and morbidity even though they are readily preventable [5–8]. Many existing child health interventions require sustained behavior change [9] (e.g. hygiene practices or care-seeking behavior) or substantial infrastructure improvements (e.g. water and sanitation) to break transmission routes [10], making implementation a challenge.

In 2015, Pakistan had the third highest mortality rate in neonates and children under 5 years and was among the ten countries with the highest under-5 mortality burden for both diarrhea and lower respiratory infection [11, 12]. At the current rates of decline in mortality [13] Pakistan will not achieve the Sustainable Development Goal 3 (SDG3) of reducing under-5 mortality to ≤25 deaths/1000 live births (LB) by 2030 [14]. Against this background, it is noteworthy that there have been successful community-level programs to improve health in some of the more remote, rural districts of Gilgit-Baltistan [15].

In 1974, the Aga Khan Health Service, Northern Areas and Chitral, Pakistan (AKHS,P) began developing a network of maternal and child health (MCH) centers in Gilgit and Ghizar districts [16, 17]. A 1986 study in the Punial Valley, west of Gilgit town, reported an infant mortality rate of 158 per 1000 LB, higher than nationwide estimates for rural Pakistan [18]. In response, AKHS,P launched an intensive primary health care program in 1987 that trained volunteer community health workers and existing traditional birth attendants, supervised by Lady Health Visitors (LHVs) [19, 20], and newly hired community-based doctors, to administer basic interventions such as health education, MCH care, immunization, treatment of common diseases, and provision of essential drugs [16, 17]. Concurrent with these health initiatives, the Aga Khan Education Service began subsidizing girls’ education in the area as early as 1969 [21], and the Aga Khan Rural Support Programme began supporting village organizations in the early 1980s [22, 23].

Following a severe dysentery outbreak in a neighboring valley, the Oshikhandass Diarrhea and Dysentery Project was initiated (1989–1996) in a rural, predominantly agricultural village 20 km southeast of the administrative capital of Gilgit and 510 km from Islamabad. Verbal autopsies showed that over half of all deaths among children younger than 5 years in this village occurred by age 4 months, with pneumonia (44%) and diarrhea (35%) being the leading causes [24]. Here we assess improvements in under-5 mortality, and diarrhea and pneumonia morbidity during this study, which included active community health education and child health interventions, and describe under-5 diarrhea and pneumonia incidence in this community 15 years later (2011–2014). We reflect on the epidemiology of common childhood illnesses in a low-resource setting with a history of community-based health interventions against a background of socioeconomic changes.

## Methods

### Data collection and protocol

Two prospective open-cohort studies were conducted in Oshikhandass village, from September 16, 1989 to September 15, 1996 (Study 1) and November 11, 2011 to March 31, 2014 (Study 2); surveillance in Study 2 was interrupted from early February until late March, 2012 due to political unrest. All children in Oshikhandass under age 60 months with parental/legal guardian consent were enrolled, including those born and migrating into the village, and followed to 60 months, out-migration, or death; the total number of children was updated continually throughout the study. Women from the village, nominated by local women’s organizations, were recruited and trained in World Health Organization (WHO) guidelines. These women visited children weekly and asked mothers whether the child had diarrhea in the past week. From December 10, 1992 to June 18, 1996 (Study 1) and January 30, 2012 to March 31, 2014 (Study 2), pneumonia was added to the study protocols and mothers were also asked about cough and difficulty breathing. During these visits study staff provided information to mothers on the causes and management of diarrhea and pneumonia and promoted breastfeeding (including exclusive breastfeeding up to 4 months in Study 1 and 6 months in Study 2), immunization, hygiene, and boiling of drinking water. During Study 2, a survey was conducted to determine mothers’ knowledge of the causes of diarrhea and pneumonia and their treatments.

Supervision in Study 1 was provided by two LHVs, a doctor, and the principal investigator of the study (ZR); and starting in December 1992, when pneumonia was added to the study protocol, study staff were available daily at the government dispensary to examine and treat children with pneumonia between study visits. In Study 2 supervision was provided by a Senior LHV and two sociologists, and study staff were not available in a central location on days outside of regular home visits. In both studies, children with at least one visit after enrollment were included for analysis. Children who died before they were visited were included for mortality, but not morbidity analysis. If a child died anytime while under follow-up, the date and cause of death were recorded from verbal autopsies.

Diarrhea was defined as having three or more loose or liquid stools per day or bloody diarrhea (dysentery), by maternal report, at any time. Diarrheal episodes were defined as consecutive days of diarrhea separated by ≥2 diarrhea-free days and categorized by duration: acute, < 7 days; prolonged, 7 to 13 days; and persistent ≥14 days. In case of diarrhea, history was taken of bloody stools, tenesmus, fever, vomiting frequency, breastfeeding status, other milk consumption, appetite, thirst and medications used; examination included general status (alert, unwell/irritable/lethargic, very sleepy/unconscious), skin turgor, temperature (axillary), rectal prolapse, and weight. Study staff provided oral rehydration packets, zinc supplements (Study 2 only), nutritional advice, antibiotics for dysentery (Study 1 cotrimoxazole; Study 2 ciprofloxacin) or referred the child if danger signs, severe dehydration or malnutrition were present.

If the mother reported the child had cough and/or difficult breathing, project staff assessed for pneumonia. Pneumonia was diagnosed as per WHO guidelines, however these changed slightly between the two studies. In Study 1, pneumonia was defined as cough with fast breathing based on age-specific cutoff breathing rates using a one-minute Acute Respiratory Infection timer [25], presence of chest indrawing (severe pneumonia), and associated danger signs (very severe disease): unable to drink, convulsions, abnormally sleepy/difficult to wake, stridor in a calm child, severe malnutrition [26]. By Study 2 danger signs had been modified to include also the inability to drink or eat anything, or vomiting everything [27]. Infants < 2 months with pneumonia were required to have immediate referral and were excluded from pneumonia rate calculations as data collection was incomplete for this age group.

Children with non-severe pneumonia were treated for 5 days with cotrimoxazole in Study 1, and with 3 days of cotrimoxazole or amoxicillin in Study 2 [28]. Children with severe pneumonia were given an initial dose of amoxicillin and referred immediately to a physician. In Study 2, children with wheezing were given up to three bronchodilator treatments via spacer or nebulizer in the study office prior to diagnosis. All children with pneumonia, regardless of severity, were seen by a supervisor the same or next day to make sure the family followed recommendations and the child’s condition had not worsened. Any child who was not improving after 48–72 h was changed to a second-line antibiotic. All children were evaluated after 5 days and again after 14 days to ensure symptoms were resolved.

In 1995, Pakistan’s Lady Health Worker (LHW) Programme [29] was introduced in the community. Of the eight LHWs selected to serve Oshikhandass, three had been trained as study staff in Study 1. LHWs continued to serve the community during the intervening years between Study 1 and Study 2. Their activities were similar, but less intensive than those of the study staff, with the additional task of providing contraceptives to women in the community [29]. Also in the intervening periods between studies, a water filtration plant was built (2002), supplying only part of the community, and *H. influenzae* type b (Hib) vaccination was initiated by the Expanded Program on Immunization (2009), given as part of a pentavalent vaccine at 6, 10, and 14 weeks of age [30].

In both studies, length/height was measured every 3 months and weight was measured monthly. Data on socioeconomic status and family characteristics were collected by questionnaire; families were defined as a mother, father and children; households were defined as multiple families which shared the same kitchen. In Study 1, questionnaires were administered initially in September 1989, new families were added as they entered the village, and questionnaires were updated every 6 months to reflect additional family members; in Study 2, family questionnaires were administered initially in November 2011 and updated to include new families until March 2014. In both studies, questionnaires were administered to all households in the village regardless of if a child was enrolled in the study. Where possible, comparable categories between Study 1 and Study 2 were used for analysis. Variables included categories of housing based on construction materials (improved: cement, concrete, brick; unimproved: no walls, mud, wood, stone; somewhat improved: a combination of the previously described categories, only used for Study 1); the number of rooms in the house and crowding (people per room). In Study 2, water from the filtration plant constructed in 2002, water brought from Gilgit, and purchased mineral water, were defined as improved drinking water sources. There were no improved drinking water sources in Study 1, but households were asked if they treated their water, which generally consisted of allowing glacier sediment to settle. Improved sanitation facilities included flush toilets (to septic tank or pit latrine) and new composting twin pit latrines; unimproved sanitation facilities included traditional pit latrines and open defecation. Improved cookstoves (Study 2) included gas or electric stoves or Building and Construction Improvement Program stoves with or without a water heater [31]; and clean cooking fuels (Study 1) included gas or electricity. Although most households reported using multiple cooking methods, the stove/fuel reported as most frequently used was taken for analysis. In Study 2 information on indoor air pollution was collected through study staff observation and self-report by the survey respondent. Information on household income was divided into quintiles and parental education was categorized in 3 levels (category 1: illiterate/ no formal education; category 2: primary – matriculation/Class 10; category 3: intermediate/12 years and above). Mothers were asked if they earned an income or not, and fathers’ occupations were divided into 4 levels based on salary and social status.

All data, including childhood health data and socioeconomic variables, were collected on paper forms by study staff and stored securely at the AKHS,P office (Study 1) and Karakoram International University (Study 2). Data were double entered and stored in SPSS (IBM, New York, USA) databases. Ethical review for Study 1 was provided by the Aga Khan University Ethical Review Committee. For Study 2, ethical review was provided by the U.S. National Institute of Child Health and Development’s Institutional Review Board, the Aga Khan University Ethical Review Committee, and the Karakoram International University Ethical Review Committee.

### Statistical analysis

Incidence and prevalence are reported in child-years (CY) at risk for diarrhea or pneumonia and described in terms of age and season. Children were considered under follow-up if the interval between visits was ≤15 days; intervals > 15 days were excluded from the observation period. CYs at risk were calculated by subtracting the number of days a child had diarrhea/pneumonia (excluding the day the episode started) from the total number of days followed and dividing by 365.25. Incidence rates were calculated by dividing the total number of episodes by the total CYs at risk and prevalence rates were calculated by dividing the total days of diarrhea or pneumonia by the total CYs of follow-up. Hurdle models [32] were constructed for each study separately to examine putative risk factors for both diseases. These models account for the large number of children with no reported episodes of diarrhea and pneumonia (zero-inflated) by separately describing the chances of never having had an episode (a logit function) and if there was a non-zero number of episodes, then how many (a Poisson function). A random intercept was included in the model to account for shared facilities between family members [33]. The hurdle models were used to predict the number of episodes/CY for a unit change for each level of the independent variables. Additionally, simulated data were used to predict the number of episodes/ CY for an idealized scenario (all improved/ highest level categories) and a reference scenario (all unimproved/lowest level categories) for both diarrhea and pneumonia during each study period. Models were constructed with the glmmTMB package [34] in R version 3.5.0 [35].

## Results

There was considerable change in the socioeconomic status of the community between the two studies including higher parental education, and improved water, sanitation, and housing materials (Table 1). The population more than doubled from approximately 3500 in 1989 to more than 7000 in 2011, however the birth rate decreased from 36 (1989–90) to 23 (2013–14) per 1000 population. Similarly, the average household size shrank from 12.3 people per house to 9.0. This coincided with a shift in occupations from predominantly manual labor and agricultural work to more entrepreneurial work such as shopkeeping and small business ownership. Despite improved maternal education and reductions in maternal illiteracy (70% in Study 1 to 28% in Study 2), over 85% of mothers were housewives in both studies. The proportion of children living in a household with an improved toilet grew from 3% in Study 1 to 65% in Study 2. There were no improved drinking water sources in Study 1 as most relied on allowing water to settle, but 33% of children in Study 2 were getting water from an improved source. Of those not getting water from an improved source in Study 2, almost 23% of children were living in a household that reported boiling drinking water. Wood was the primary cooking fuel in both studies and use of improved cookstoves in Study 2 was rare (1.8%). In Study 2, 29.4% of children were exposed to visible smoke in the house based on study staff observation at the time of interview. Additionally, 83.2% of children were living in a household where the survey respondent thought there was smoke in the home in the evenings during stove use.

**Table 1:** Description of changes in socioeconomic and demographic characteristics between Study 1 and Study 2

	Study 1	Study 2
Children younger than 5 years followed	1843	1169
Median age in months of children in the study during follow-up (IQR)	28.8 (14.3–43.8)	28.7 (14.0–44.5)
Female sex (%)	879 (47.7)	557 (47.6)
Families with children followed in the study^#^	649	675
Households with children followed in the study ^#^	433	572
Mean household crowding (SD)	7.9 (4.7)	3.6 (2.2)
Children in improved houses (%)	389 (21.1)	636 (54.5)
Children in households using clean cooking fuel*; improved cookstove** (%)	45 (2.4)	21 (1.8)
Children living in house with improved toilet (%)	48 (2.6)	767 (65.7)
Children in household that settles drinking water*; has improved drinking water source** (%)	992 (53.8)	386 (33.0)
Children with illiterate mother (%)	1303 (70.7)	330 (28.2)
Children with illiterate father (%)	562 (30.5)	127 (10.9)
Median Monthly Household Income in Pakistan Rupees (Incomes at 2010 prices)	2999 (18325)	25,000 (20367)
< 5 mortality/1000 live births (total deaths)	86.8 (103)	19.8 (8)
Infant mortality/1000 live births (total deaths)	71.7 (85)	7.4 (3)

^#^ Multiple families could share a household. Families were defined as a mother, her husband and her children. Households were defined as multiple families sharing the same kitchen

* Study 1 **Study 2

In Study 1, 1843 children younger than 5 years were followed and 1169 were followed in Study 2, of whom 73% belonged to a household enrolled in the first study. The under-5 mortality rate oscillated from year to year over the course of Study 1 but exhibited a significant downward trend (linear regression weighted by months of surveillance, mean deaths/1000 LB per year: -3.2, 95%CI − 1.7 to − 4.8, Table 2). Between Study 1 and Study 2, the under-5 mortality rate decreased from 86.8 to 19.8 deaths/1000 LB, and the infant mortality rate decreased from 71.7 to 7.4/1000 LB (Table 1). Most of the deaths in Study 1 (85/103, 82.5%) occurred in infancy, with a median age at time of death of 3.6 months and a quarter (26/103) within the first month of life; none occurred in children older than 48 months. The leading cause of death was diarrhea (34/103, 33%), followed by pneumonia (24/103, 23%), however neither were observed as a cause of death in children over 18 months. Mortality was distinctly seasonal with 82.3% of deaths from diarrhea occurring during summer months (May–September) and 54.2% of deaths from pneumonia occurring in winter months (October–February). In Study 2, only eight deaths occurred, all in children less than 36 months, with three in infants less than 12 months. There was one death from diarrhea and no deaths due to pneumonia.

**Table 2:** Morbidity and mortality incidence and treatment by year

	1989^a^	1990	1991	1992	1993	1994	1995	1996^a^	2011^a^	2012^a^	2013	2014^a^
< 5 Mortality/ 1000 LB	114.3	129.9	69.0	57.3	102.0	73.0	74.9	79.5	0	22.9	16.1	50.0
Diarrhea episodes	291	587	453	351	401	294	240	211	107	423	589	26
Diarrhea incidence/ CY (95%CI)	2.1 (1.9–2.4)	1.1 (1.0–1.2)	0.8 (0.7–0.9)	0.6 (0.6–0.7)	0.6 (0.6–0.7)	0.5 (0.4–0.5)	0.4 (0.3–0.4)	0.5 (0.4–0.5)	1.3 (1.0–1.5)	0.7 (0.6–0.8)	0.8 (0.7–0.9)	0.2 (0.1–0.2)
Diarrhea prevalence/ CY (95%CI)	20.3 (19.6–21.1)	7.0 (6.8–7.2)	6.3 (6.1–6.5)	4.8 (4.6–4.9)	4.8 (4.6–4.9)	3.0 (2.9–3.1)	2.4 (2.3–2.5)	2.2 (2.1–2.4)	7.3 (6.7–7.8)	2.5 (2.4–2.6)	2.7 (2.6–2.9)	0.4 (0.3–0.5)
Bloody diarrhea (%)	54 (18.6)	68 (11.6)	56 (12.4)	50 (14.2)	45 (11.2)	47 (16.0)	19 (7.9)	11 (5.2)	5 (4.7)	22 (5.2)	33 (5.6)	2 (7.7)
Acute diarrhea (%)	108 (37.1)	371 (63.2)	244 (53.9)	182 (51.9)	243 (60.6)	180 (61.2)	151 (62.9)	168 (79.6)	76 (71.0)	393 (92.9)	567 (96.3)	25 (96.2)
Given Extra fluids by mother (%)	227 (78.0)	473 (80.6)	392 (86.5)	324 (92.3)	324 (80.8)	245 (83.3)	189 (78.8)	187 (88.6)	32 (29.9)	187 (44.2)	285 (48.4)	11 (42.3)
Given ORS by mother (%)	123 (42.3)	303 (51.6)	297 (65.6)	242 (68.9)	198 (49.4)	164 (55.8)	123 (51.3)	112 (53.1)	26 (24.3)	151 (35.7)	216 (36.7)	5 (19.2)
Given ORS by RW/ LHW (%)	269 (92.4)	491 (83.6)	397 (87.6)	316 (90.0)	270 (67.3)	173 (58.8)	169 (70.4)	88 (41.7)	36 (33.6)	329 (77.8)	469 (79.6)	24 (92.3)
Prescribed antibiotics (diarrhea) (%)	28 (9.6)	48 (8.2)	20 (4.4)	2 (0.6)	4 (1.0)	2 (0.7)	1 (0.4)	1 (0.5)	5 (4.7)	45 (10.6)	31 (5.3)	3 (11.5)
pneumonia episodes				18^a^	312	208	303	150		38^a^	199	19
Pneumonia Incidence / CY (95% CI)				0.5 (0.3–0.7) ^a^	0.5 (0.5–0.6)	0.3 (0.3–0.4)	0.5 (0.4–0.6)	0.5 (0.4–0.6)		0.1(0.1–0.1)^a^	0.3 (0.2–0.3)	0.1 (0.1–0.2)
Pneumonia prevalence/ CY (95% CI)				2.7 (2.2–3.3) ^a^	2.9 (2.7–3.0)	1.9 (1.8–2.0)	2.9 (2.8–3.1)	3.0 (2.8–3.2)		0.2 (0.2–0.3) ^a^	1.9 (1.8–2.0)	0.8 (0.7–1.0)
Severe pneumonia or very severe disease (%)				0 (0.0) ^a^	23 (7.4)	8 (3.8)	4 (1.3)	12 (8.0)		1 (2.6) ^a^	11 (5.5)	1 (5.3)
receiving antibiotics for pneumonia from non-study source (%)				0 (0.0) ^a^	28 (9.0)	21 (10.1)	20 (6.6)	11 (7.3)		22 (56.4)	67 (33.7)	3 (15.8)

^a^Not a full calendar year of surveillance. Please see methods section for details on dates and months of follow-up

**Fig. 1:**
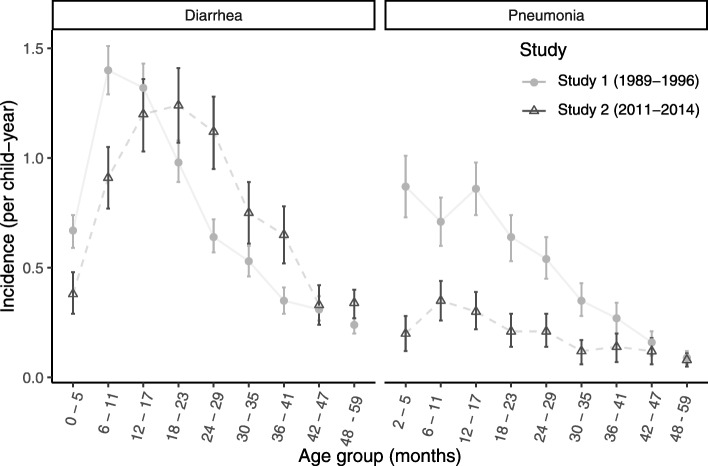
Incidence of diarrhea and pneumonia by age group for each study period

**Fig. 2:**
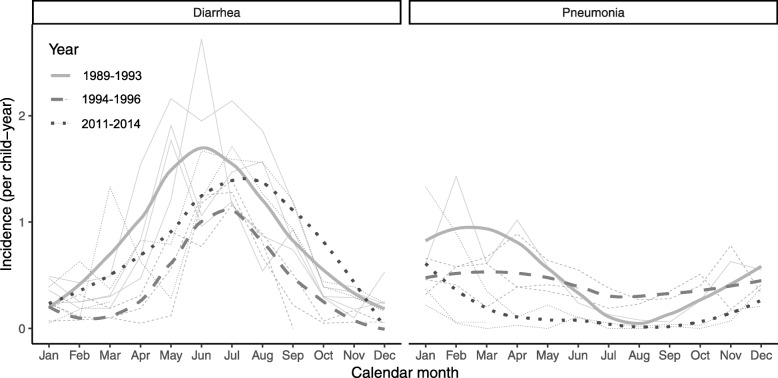
Incidence of diarrhea and pneumonia by month and year

During Study 1, the incidence of diarrhea was 0.7 episodes/ child-year (CY) and the prevalence was 4.8 days/CY, with the highest incidence in children 6–11 months (Fig. 1). Diarrheal incidence decreased over the 7-year study period from 2.1 episodes/CY in 1989 to 0.5 episodes/CY in 1996; although incidence was higher in the summer months in both periods, the peak shifted from June to July after 1993 in Study 1, and this trend continued in Study 2 (Fig. 2). In Study 1, 61.7% of children had an episode of diarrhea while under follow-up. The majority (58.2%) of diarrhea episodes were acute (mean duration 7.1 days), and 12.4% of episodes were bloody (Table 2). The proportion of episodes in Study 1 receiving oral rehydration solution (ORS) from their mother increased in the third year of the study (from an initial 42.3 to 68.9%) and then plateaued (around 50%), while the percentage receiving extra fluids remained high at over 78%.

In Study 2, diarrhea incidence was also 0.7 episodes /CY, but prevalence decreased to 2.7 days/ CY and incidence peaked in older children (18–23 months) (Fig. 1). Only 43.1% of children had an episode of diarrhea while under follow-up, 93% of episodes were acute (mean duration was 3.7 days), and 5.4% of episodes were bloody. ORS was given by mothers to only 34.8% of episodes; however, 94% of mothers believed that ORS was a remedy for diarrhea and over 60% indicated that they were familiar with how to prepare ORS at home. Study staff provided ORS packets for 74.9% of episodes. Overall, antibiotics were given by the study staff for 3.7% of episodes (61.3% of which were recorded as bloody) in Study 1, but this rose to 7.3% of episodes (33.3% of which were reported as bloody) in Study 2 (Table 2). In Study 1, mothers reported breastfeeding during 80.8% of episodes in children < 24 months and 89.9% of episodes in children < 12 months (exclusive breastfeeding in 55.5% of those < 4 months). In Study 2, mothers reported breastfeeding during 70.3% of episodes in children < 24 months, and 88.3% of episodes in children < 12 months (exclusive breastfeeding in 44.6% those < 6 months).

Pneumonia incidence was 0.5 episodes/CY with a prevalence of 2.6 days of pneumonia/CY, with peak incidence in those 2–5 months in Study 1. By Study 2 the incidence was substantially lower (0.2 episodes/CY) and prevalence halved (1.1 days/CY), with peak incidence in those 6–11 months (Fig. 1). In Study 1, 41.2% of children had an episode of pneumonia while under surveillance, whereas only 17.8% did in Study 2. Incidence tended to be higher in the winter months, but there were no statistically significant trends in seasonality (Fig. 2). The proportions of children diagnosed with severe pneumonia (3.7% versus 3.9%) or very severe disease (1.0% versus 1.2%) were similar between the two study periods (Table 2). In Study 2, 35.8% of children with pneumonia had received an antibiotic from other (non-study) sources before the study examined them, compared to only 8.1% in Study 1.

In Study 1, 1515 children had at least one length/height measurement available, of which 1226 (80.9%) had at least one Length-for-age z-score (LAZ) < − 2, compared to Study 2 in which 409 of 989 (41.1%) children with length/height measurements available had at least one LAZ < -2 (*p* < 0.0001). Among children with a length/height measurement available at 24 months (±3 months), the mean LAZ was − 2.7 (SD 1.3) in Study 1 (749 children) and − 1.6 (SD 1.2) in Study 2 (280 children) (*p* < 0.0001). Birthweight was not collected, but for children with a weight measurement in the first 3 months of life, average weight in kilograms was 4.6 (SD 1.0) in Study 1 (375 children) and 4.8 (SD 1.6) in study 2 (329 children) (*p* = 0.05).

**Fig. 3:**
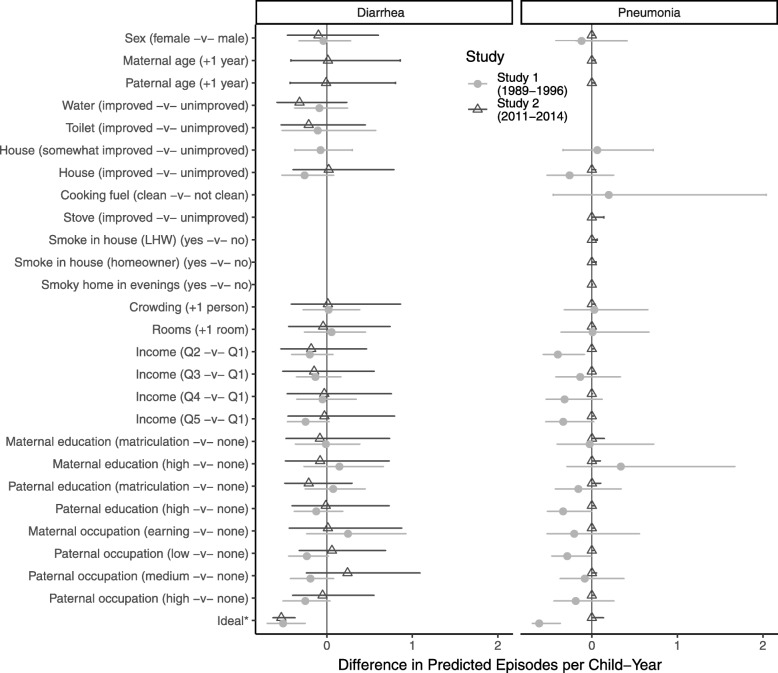
The predicted difference from the reference scenario (all unimproved/lowest categories) in the number of episodes of diarrhea and pneumonia comparing putative risk factor levels for each study period

In the multivariable analysis using hurdle models no individual factor predicted a statistically significant difference in number of diarrhea episodes (at *p* ≤ 0.05, Fig. 3), although both improved water and toilet tended to be protective (associated with having no episodes and fewer episodes in the hurdle model, respectively) and their combined effect was a significant reduction in predicted episodes. The predicted incidence of diarrhea for the reference scenario was 0.9 (0.6–1.3)/CY in Study 1 and 0.7 (0.3–1.5)/CY in Study 2, whereas the predicted incidence for a simulated idealized scenario was 0.4 (0.2–0.7)/CY in Study 1 and 0.2 (0.1–0.4)/CY in Study 2. In Study 1 the single most protective factor was living in a house constructed with higher quality materials (predicted incidence 0.7 (0.4–1.0)/CY), and in Study 2 the single most protective factor was getting water from an improved source (0.4 (0.1–0.9)/CY).

Given the small number of episodes per CY, factors associated with pneumonia tended to have small incremental effects, especially in Study 2. The predicted episodes under the reference scenario for Study 1 was 0.7 (0.4–1.3)/CY) compared to the idealized scenario which was 0.1 (0.02–0.4)/CY) with income, higher status parental occupations, and more paternal education being the most protective factors (Fig. 3). In Study 2, however, there was no notable difference between the idealized and reference scenarios, and no individual factors were associated with the predicted number of episodes (Fig. 3).

## Discussion

Despite its remote location and difficult terrain, there is lower child mortality in Oshikhandass than estimates for Pakistan as a whole [13], even exceeding the SDG3 targets [14]. Indeed, the progress achieved early during Study 1 was sustained, with a 4-fold reduction in under-5 mortality and more than nine-fold decrease in infant mortality, 15 years later. This decrease was similar to, but greater than the 43% reduction of under-5 mortality (70 to 30 deaths per 1000 LB) or two-thirds reduction in infant mortality (60 to 20 deaths per 1000 LB) in Pakistan’s Northern Areas between 1992 and 2008 [16].

Although the incidence of diarrhea was similar in both studies, in Study 2 episodes were half as long and fewer were bloody, which suggests fewer episodes of bacterial diarrhea [36]. However, because diarrhea episodes were still concentrated in the summer months, which tends to be associated with bacterial etiologies, it is likely that diarrhea is still predominantly bacterial [37]. A larger than expected proportion of children never had an episode of diarrhea while under surveillance. This was true for both studies, but especially for Study 2 and might be partially explained by the shorter follow-up period compared to Study 1. This could also explain why such a small proportion of children in Study 2 had episodes of pneumonia. While there were more pneumonia cases in the winter months in both study periods, there was no statistically significant seasonal variation. Pneumonia was persistent year-round in Study 1, perhaps due to the altitude (1500 m) as suggested by other studies in similar environments [38]. Pneumonia incidence decreased substantially, dropping 60% between the two study periods. The age profile of children with diarrhea or pneumonia episodes shifted to older age groups in Study 2, consistent with decreased pathogen exposure. Delaying the peak incidence moves infections away from the most vulnerable infants, which likely drives the decreased mortality. Anthropometry data were sparse, however, childhood nutritional status improved by Study 2 based on the proportion of stunted children and mean LAZ at 24 months. Improved nutritional status is likely to have contributed to the decreased mortality and morbidity, avoiding the ‘vicious circle’ of malnutrition [39].

Repeated interactions with health workers and health and hygiene education may have also contributed to this success. In Study 1, regular home visits from study staff and their visible presence in the community spanned 7 years, and there was intensive supervision of study staff, maintaining a high level of vigilance in the community and ensuring that the integrity of interventions was preserved [40]. Furthermore, 73% of children in Study 2 were living in a household that had participated in Study 1 and this may have helped preserve knowledge and experience in the community-base and ensured that interventions had a lasting impact. A systematic review of 24 studies showed that community-based interventions were associated with increased care seeking for diarrhea and pneumonia as well as increased use of ORS and decreased use of unnecessary antibiotics [41]. In this population we found around a 20% drop in the use of ORS by mothers during diarrhea episodes between the 2 studies, despite high levels of knowledge about its importance and how to make it at home. It may be that shorter episodes were not deemed severe enough to require ORS, or that the ORS given by study staff, which remained high (74.9%), was deemed enough. Throughout Gilgit-Baltistan, a similarly high proportion (76.8%) of children received some form of oral rehydration for diarrhea, higher than any other region in Pakistan [42]. Cereal-based ORS had been introduced in Study 1 but use of this measure was not separately collected in Study 2. Conversely, the access to and use of antibiotics increased between studies despite an initial drop in their use during Study 1. Increased familiarity with the symptoms and treatment for pneumonia may explain why such a high proportion of episodes sought treatment prior to examination by the study team in Study 2; however, it could also be that study staff were not available to examine and treat children who became sick between home visits as they were in Study 1. Future studies should include provision of a centralized point of care that includes a room for prompt diagnoses to ensure accurate surveillance and timely treatment.

The combined effect of improved socioeconomic factors and water and sanitation was associated with fewer disease episodes. The physical environment (housing materials, flush toilets, and access to clean water) tended to be more strongly related to the risks of diarrhea, whereas socioeconomic variables (education, occupation, and income) determined risk of pneumonia in Study 1, with pneumonia concentrated in the poorest households. Access to the water filtration plant in Study 2 was determined by location in the village; this was a substantive infrastructural undertaking, but it was not dependent on families’ individual socio-economic status. In the first study, very few households exclusively used a flush toilet, and while people reported treating drinking water, generally this meant that glacier water with high silt was allowed to settle. In Study 2, about a quarter of those not getting water from an improved water source boiled their drinking water. This likely had a protective effect against diarrhea which was not accounted for in the hurdle model.

Vaccination is an important strategy for reducing diarrhea and pneumonia burden in children. A randomized-controlled trial in Pakistan demonstrated increased vaccination coverage following educational interventions provided by community health workers [43]. *H. influenzae b* vaccination has been routinely given in Oshikhandass since 2009, and this is believed to have contributed to the significant decline in pneumonia rates, as evidenced in other studies [44, 45]. Similarly, the Pneumococcal conjugate vaccine, introduced in 2012, is likely to further reduce pneumonia incidence. The rotavirus vaccine, the only vaccine in this population specifically targeting a common cause of childhood diarrhea, was introduced in Oshikhandass in July 2018 (personal communication through ZR) and is expected to drive a reduction in diarrhea over the next few years, which will be important for future research in this community.

This study focuses on a unique population living in a remote area of a country with some of the most persistently high rates of childhood morbidity and mortality. A notable strength of this is study is that despite the long timespan of this analysis (1989–2014), about a third of the study staff were involved in both the initial study and more recent follow-up and were able to collect comparable information during both periods. However, the supervision and support given to the study staff was more comprehensive in Study 1, which likely had a positive impact on health worker performance as suggested by other studies [40, 46]. Similarly, in Study 1 community members could more easily meet with a member of the study team if their child became sick in between visits, but in Study 2 were more likely seek care elsewhere. Other strengths include using local community members as study staff, which promoted sustainability and community acceptance of study activities, and a study design that was responsive to changing WHO definitions and guidance, for example adding pneumonia surveillance when it became apparent this was needed.

It is important to note the substantial socio-demographic changes between the two studies. Although aspects of this were captured with changing patterns of education, employment and access to water and sanitation, other important indicators were not recorded, for example, hygiene practices, household and community waste management, indicators of maternal health, preterm births, birthweight, and nutritional indicators. There may also be other unidentified potential confounders. The rate of breastfeeding through diarrheal episodes was high in both studies, but exclusive breastfeeding to 4 months in Study 1 and 6 months in Study 2 was low. Unfortunately, these data were only collected as part of the diarrhea questionnaire, so it was not possible to compare risk for diarrhea based on breastfeeding status, and no other food intake data were collected. Over the course of the studies, changing patterns of infant and young child feeding practices may have supported reductions in morbidity and mortality. Improvements in nutritional status are implied in the improved average LAZ of children, but again these data were sparsely collected given the different ages of children enrolled. Increased access to contraception and family planning services may have contributed to the decreases in birth rate and household size. This, combined with the observed occupational changes, likely resulted in more resources per person at the household level, which we expect supported improvements in child health.

Additional limitations to the study design include that pneumonia surveillance was added to the protocol later in both studies, which reduced our ability to detect patterns, and the scarcity of clean fuel or stoves, while of itself interesting and a potential opportunity to further reduce pneumonia rates, was too low to permit meaningful analysis. Surveillance was incomplete for some calendar years and consequently some months had fewer observations to estimate seasonal variation. Further, due to political unrest, surveillance is incomplete between early February 2012 and late March 2012, and this could have an impact on incidence estimations of pneumonia but is less likely to affect diarrhea incidence.

## Conclusions

We report a 4-fold decrease in overall childhood mortality, and a 2-fold decrease in childhood morbidity from diarrhea and pneumonia in a remote rural village in Pakistan. Individual factors were not consistently associated with the risks of diarrhea or pneumonia, however the combined improvements to housing materials, sanitation and water supply were predictive of lower incidence of diarrhea. In Study 1, higher household incomes, employment, and paternal education tended to reduce pneumonia incidence, but there were too few episodes in Study 2 to gauge the impact of these variables. We expect that vaccination with Hib vaccine after 2008 contributed to this reduction in pneumonia. However, despite intense health education, there has also been an increase in antibiotic use and a decrease in ORS use for diarrhea. We conclude that significant, sustainable improvements in child health may be achieved through improved socioeconomic status and promoting interactions between locally engaged health workers and the community, but that continued efforts are needed to improve health worker training, supervision, and the rational use of medications..

## Data Availability

Data are available from the corresponding author (ZR) upon reasonable request.

## Abbreviations

AKHS,P: Aga Khan Health Service, Northern Areas and Chitral, Pakistan
CY: Child-years
Hib: *Haemophilus influenzae* type b
LB: Live births
LAZ: Length-for-age z-score
LHV: Lady Health Visitor, women with ≥12 years of education and 2 years of education in public health and midwifery at government institutions
LHW: Lady Health Worker, women with ≥8 years of education and 15-months of training (3 months of classroom instruction and 12 months of practical training)
MCH: Maternal and Child Health
ORS: Oral Rehydration Solution
SDG3: Sustainable Development Goal 3
WHO: World Health Organization
